# Mechanosynthesis of High-Nitrogen Steels Strengthened by Secondary Titanium Nitrides

**DOI:** 10.3390/ma15145038

**Published:** 2022-07-20

**Authors:** Valery Shabashov, Kirill Lyashkov, Andrey Zamatovskii, Kirill Kozlov, Natalya Kataeva, Evgenii Novikov, Yurii Ustyugov

**Affiliations:** 1 M.N. Mikheev Institute of Metal Physics, Ural Branch, Russian Academy of Sciences, 620108 Ekaterinburg, Russia; lyashkov@imp.uran.ru (K.L.); zamatovsky@imp.uran.ru (A.Z.); kozlov@imp.uran.ru (K.K.); kataeva@imp.uran.ru (N.K.); evg_nov@mail.ru (E.N.); ustyugov@imp.uran.ru (Y.U.); 2 Institute of Engineering Science, Ural Branch, Russian Academy of Sciences, 620108 Ekaterinburg, Russia

**Keywords:** mechanical alloying, high-nitrogen steels, titanium nitrides, atomic redistribution, Mössbauer spectroscopy

## Abstract

The solid-phase mechanical synthesis of high-nitrogen ferritic and austenitic steel composites in the course of mechanical activation in a ball mill is studied by the method of Mössbauer spectroscopy and electron microscopy. For mechanical alloying, mixtures of iron alloys doped with transition metals (Ni, Cr, Mn, and Ti) and nitrides with low stability to deformation (CrN and Mn_2_N) were used. The correlation between the phase–concentration composition of the mechanically synthesized samples and the heat of formation of transition metal nitrides, which are part of the initial metal mixtures, is investigated. It is established that the use of titanium as an alloying additive of the Fe component of the mixture accelerates the processes of dissolution of primary nitrides and allows the transference of chromium and manganese to the position of substitution in the metallic solid solution. In addition, the titanium additive entails the formation of secondary nitrides with stabilizing the nanostructure of the mechanically synthesized samples.

## 1. Introduction

Iron-based alloys doped by the transition metals Ni, Co, Mn, V, and Ti and the interstitial chemical elements C, N, B, and O are the base for the development of new technologies using the dispersion hardening and nanostructuring of steels in mechanical engineering. The same types of electron structure and the closeness of values of atomic radii of 3*d* transition metals are the conditions for the formation of metallic solid substitution solutions with a large range of mutual solubility, high melting point, and different symmetry of the crystal lattice (BCC, FCC, HCP, etc.). Physico-mechanical properties are characterized by the large values of binding forces between atoms and polymorphism, heat resistance, corrosion resistance, of alloys, etc. In practice, alloying with interstitial chemical elements (C, N, B, and O), which leads to creation of the interstitial solid solutions and interstitial phases, is of great importance for improving the operational properties of iron alloys. The interstitial phases are characterized by stronger bonds between the atoms, namely, covalent, ionic ones, which makes these phases useful for creating natural composites with a strengthening dispersed additive in the form of carbides, nitrides, oxides, etc. The choice of nitrogen for the alloying iron is due to the possibility of creating high-strength composite steels hardened with thermally stable nitrides (TiN, AlN, ZrN, etc.) [1,2,3,4]. At present, there are several methods for the nitrogen alloying of steels, among which the main one is smelting with nitrogen back pressure [2]. The great interest in nitrogen is also due to the possibilities of modifying the surface layers of steels using ion-plasma nitriding [5,6,7] and technologies with intensive deformation and nanostructuring [8,9,10,11,12,13]. An important role in the creation of metal composites is played by mechanical alloying (or mechanosynthesis), which allows the formation of supersaturated solid solutions with the subsequent precipitation of hardening secondary phases. In connection with the development of technologies using mechanical alloying, a more economical method of solid-phase mechanical nitrogen alloying of iron alloys was proposed in [13,14,15,16,17]. The peculiarity of the proposed method of solid-phase mechanical nitriding is the use of nitrides that are unstable during plastic deformation for their mechanical introduction into a metal matrix, i.e., the process of mechanical alloying (MA) of the latter by nitrogen. A similar approach was implemented to obtain ODS alloys, where iron and copper oxides, unstable during deformation, were used as oxygen donors [18,19]. In the MA process, or in other words, in the process of mechanical synthesis (MS), along with the deformation-induced dissolution of the nitride and oxide particulates, the dispersion hardening of steels occurs due to dynamic aging and the formation of secondary nano-scale nitrides [18,19,20,21] and oxides [22,23,24,25].

Solid-phase MS was performed using large plastic deformation in ball mills [14,15,16,19], under frictional action [17] and shear under pressure in Bridgman anvils [17,18], where Fe_4_N, CrN (Cr_2_N), Mn_2_N, and BN nitrides poorly resistant to deformation were used as nitrogen donors, and α-iron with additional alloying by transition elements (Ni, Cr, and Mn) was used as metal matrix. In the case of frictional action with dry sliding friction, alloying was carried out by the mechanical dissolution of nitrides on the surface of alloys [17,18].

In solid-phase mechanical synthesis, the non-equilibrium dissolution of nitride particulates and the mechanical alloying of the metal matrix take place with elements that are constituent parts of nitrides (Fe, Mn, Cr, and N) with the formation of supersaturated interstitial solid solutions and the subsequent formation of secondary phases. In [15,19,21], the influence of the physicochemical properties of the elements of the mixtures that constitute the metal matrix and nitrides on the chemical and phase composition of the products of mechanical synthesis was shown.

A special task of the solid-phase mechanical synthesis of nitrogen-containing steels is the creation of high-nitrogen austenite. The goal is in replacing expensive nickel with nitrogen, which stabilizes the high-temperature γ phase [19,21,26]. Studies have been carried out on the solid-phase mechanical synthesis of high-nitrogen austenitic chromium-nickel and chromium-manganese steels [19,21]. By the methods of Mössbauer and electron microscopy, it has been found that, during mechanical activation, supersaturated solid nitrogen-enriched solutions with BCC and FCC crystal lattices are formed in a ball mill. However, both in the process of mechanical action and as a result of subsequent annealing, the solid solutions decompose with the release of alloying elements from the metal matrix, which leads to the destabilization of austenite. In [21], the effect of austenite-forming manganese on the results of the stabilization of the mechanically synthesized structure of the γ-phase in the mixtures of Fe–*x*Mn + CrN and Fe–*y*Cr + Mn_2_N was investigated. It was found that, with the same ratio of chromium, manganese, and nitrogen elements in the compositions used, the mechanical synthesis of the second mixture leads to a larger volume of stable austenite. The stabilization of austenite in the mixture Fe–*y*Cr + Mn_2_N was promoted by the preservation of austenite-forming manganese and its heterogeneous distribution in the metal matrix of MS samples. Secondary nitrides are formed mainly in the form of chromium mononitride, due to the higher chemical activity and diffusion mobility of chromium compared to manganese. Thus, in order to control the MS processes and stabilize the emerging structure, it is necessary to take into account the physico-chemical properties of the elements of mixtures involved in mechanosynthesis.

The physico-chemical properties of the metals that constitute mechanical mixtures are determined by their electronic structure and position in the periodic system of elements. The transition elements Cr, Mn, Fe, Ni, and Ti belonging to groups IV–VIII of the periodic system form with nitrogen the nitrides. The most durable nitrides are formed by titanium, which belongs to group IV of the periodic table. Titanium nitrides have the highest heat of formation (HF); such HF characterizes the bonding forces of the metal with nitrogen. This makes titanium nitrides a promising hardening phase in steel composites [4,27,28].

In this paper, the aim is to study the effect of a titanium alloying additive in the matrix of metallic iron during mechanical synthesis with the nitrides CrN (Cr_2_N) and Mn_2_N. In addition, the aim of the work is to analyze the feasibility of the mechanical synthesis (MS) in a ball mill of the ferritic-martensitic and austenitic steel composites reinforced with disperse titanium nitrides.

## 2. Experimental Procedure

Powders of the Fe–*y*Ti binary alloys (*y*, wt.% = 2; 5) with a BCC crystal lattice were employed in the capacity of metal matrices for the MS of high-nitrogen steels. The nitrogen donor was CrN (Cr_2_N) together with Mn_2_N, which are nitrides poorly resistant to deformation; they were taken in the proportion of 20 wt.% of the mixture. The mixtures Fe–*x*Ni (*x*, wt.% = 0; 6) + CrN (Cr_2_N) were used in the capacity of model systems.

The following mixtures were analyzed (Table 1):

“A”: = Fe–2Ti + CrN;
“B”: = Fe + CrN;
“C”: = Fe–6Ni + CrN;
“D”: = Fe–5Ti + Mn_2_N.

**Table 1:** Mixture type, composition formula, content of alloying elements, and amount of austenite in the MS alloys after milling and subsequent anneals.

Formula of Composition	PBM 10 h			Annealing at 500 °C, 4 h after PBM		
	Amount of Austenite vol.%	Content of Nitrogen in Austenite, *c*_N_, at.%	Content of Alloying Metal in Ferrite, *c*_Me_, at.%	Amount of Austenite vol.%	Content of Nitrogen in Austenite, *c*_N_, at.%	Content of Alloying Metal in Ferrite, *c*_Me_, at.%
Fe–2Ti + CrN	0	–	*c*_Ti + Cr_ = 6.2	0	–	*c*_Cr_ = 5.0
Fe + CrN	7	9	*c*_Cr_ = 3.9	0	–	*c*_Cr_ = 1.7
Fe–6Ni + CrN	10	7	*c*_Cr_ = 4.6	9	2	*c*_Cr_ = 0.8
Fe–5Ti + Mn_2_N	>70	1.3	*c*_Ti + Mn_ = 12	>70	0.5	*c*_Mn_ = 4.1
			*c*_Mn_ = 10			

The choice of compositions was based on the data of the phase diagram and on the problem of obtaining the α and γ nitrogen-containing solid solutions [29,30]. In particular, it was taken into account that Cr and Mn form solid substitution solutions with iron with a BCC crystal lattice, and Ni and Mn form solid solutions with an FCC crystal lattice. Binary alloys were smelted, homogenized, and then filed with a needle-point file. The resulting powders were mixed with nitrites. Nitrides were synthesized using the technology of self-propagating thermal synthesis [31]. According to X-ray diffraction analysis, chromium nitrides were a mixture of 80% CrN + 20% Cr_2_N [32], and manganese nitrides were 80% Mn_2_N mixed with nitrides having an increased manganese content. According to the results of the chemical analysis, the synthesized manganese nitrides contained 9.1 wt.% (28.3 at.%) of nitrogen [16]. Mechanical «fusion» synthesis was carried out using a planetary ball mill (PBM) “Pulverisette-7”. The rotation speed of the mill platform was 800 rpm. The vessel for milling (container) and balls were made of high-strength ball bearing steel containing 1.5 wt.% Cr and 1.0 wt.% C; the rest was iron. The ratio of the weight of the balls (15 balls with a diameter of 10 mm) and the powder sample was 6 to 1. After loading the balls and powder mixtures, air was pumped out of the working volume to 10^−3^ mm Hg and the container was filled with an inert gas argon. The time of milling was varied from 5 to 10 h. The temperature of the outer side of the milling vessel did not exceed 70 °C. The average size of particulates of the initial powders of alloys were in the order of 200 µm, and the nitride powders were in the order of 1 µm. After termination of milling, part of the powders was annealed in a vacuum of ~10^−5^ mm Hg, at temperatures of 500–800 °C, for 1–4 h. The possible contamination of the studied samples with wear products was controlled by weighing the weight of the vessels, balls, and powder before and after mechanical alloying. The difference in the weight of the powders did not exceed the value of 0.3–0.5 wt.%. The method of assessing contamination by the weight of the vessels, balls, and powder is not absolutely deprived of, i.e., does not exclude, «errors». However, in this article, taking into account the same conditions of exposure to different mixtures of mechanical treatment and assuming the same systematic errors (in terms of the degree of contamination), differences in the results of mechanical alloying of mixtures can be considered correct. According to the results of the chemical analysis, in all cases of milling in a ball mill, the oxygen concentration in the samples did not exceed 0.8 wt.%.

The Mössbauer measurements of the MS results and subsequent annealing of powders were carried out at room temperature on an MS-1101 spectrometer with a ^57^Co(Rh) source. The spectra were calibrated using an α-Fe absorber at room temperature. The structure and the phase composition of the MS powder mixture were studied using a JEM-200CX transmission electron microscope with analysis of micro-diffraction patterns and dark-field images.

## 3. Results of the Experiments

Based on the objectives of the work, two tasks were solved: implementation of both (1) mechanical synthesis of the Fe–Cr–Ti–N ferrite-martensitic steel reinforced with titanium nitrides and (2) the mechanical synthesis of the Fe–Mn–Ti–N austenitic steel reinforced with the same titanium nitrides.

### 3.1. Mössbauer Analysis of the Phase and Concentration Composition of the Mechanically Synthesized Steels

**Figure 1:**
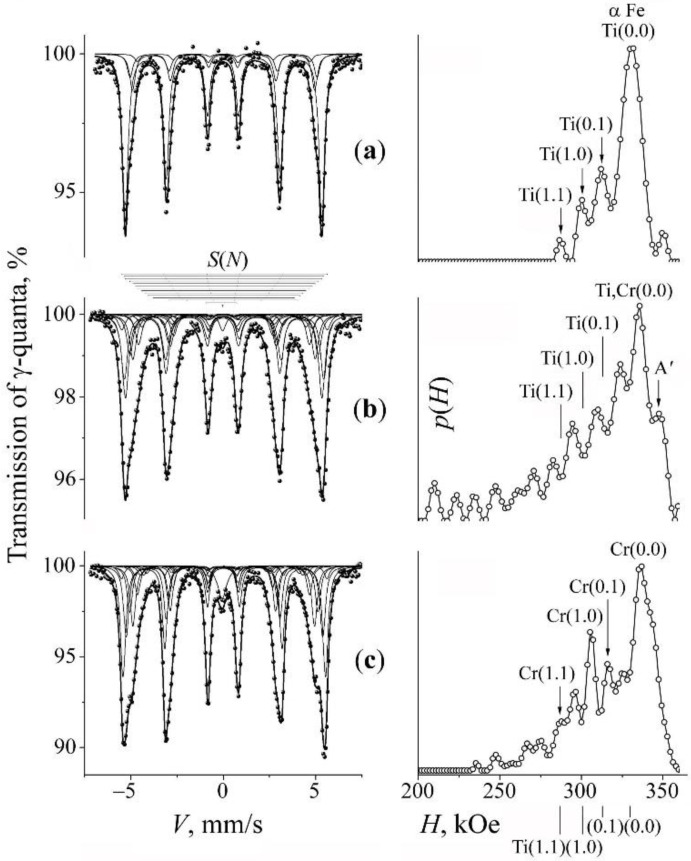
Mössbauer spectra of the mixture “A” (Fe–2Ti + CrN) and the distributions *p*(*H*) for the α-phase. Treatment: (**a**)—initial mixture; (**b**)—PBM, 10 h; (**c**)—PBM, 10 h + anneal 500 °C, 4 h. The arrows at the distribution *p*(*H*) mark the peaks corresponding to occupations by the impurity atoms Ti and Cr in the first two CSs of iron.

**Figure 2:**
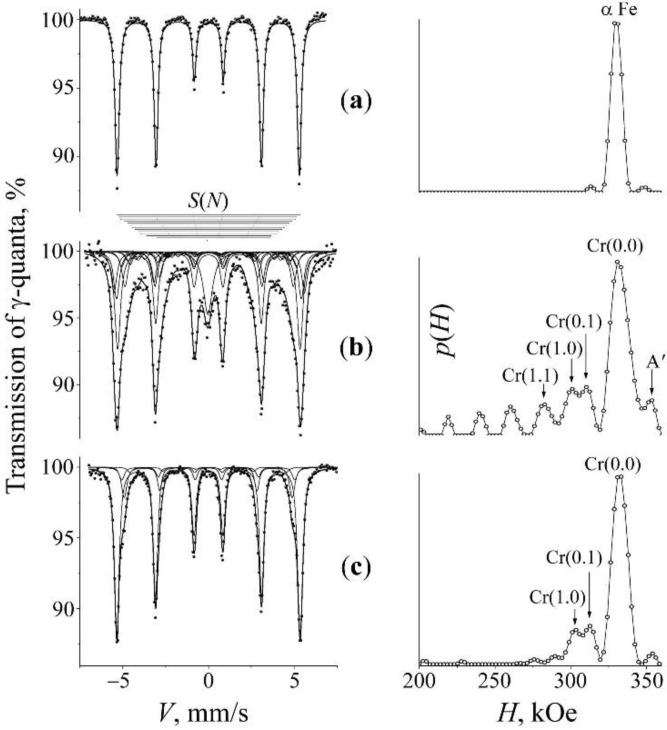
Mössbauer spectra of the mixture “B” (Fe + CrN) and the distributions *p*(*H*) for the α-phase. Treatment: (**a**)—initial armco-Fe; (**b**)—PBM, 10 h; (**c**)—PBM, 10 h + anneal 650 °C, 5 h. The arrows at the distribution *p*(*H*) mark the peaks corresponding to occupations by the impurity atoms Cr in the first two CSs of iron.

**Figure 3:**
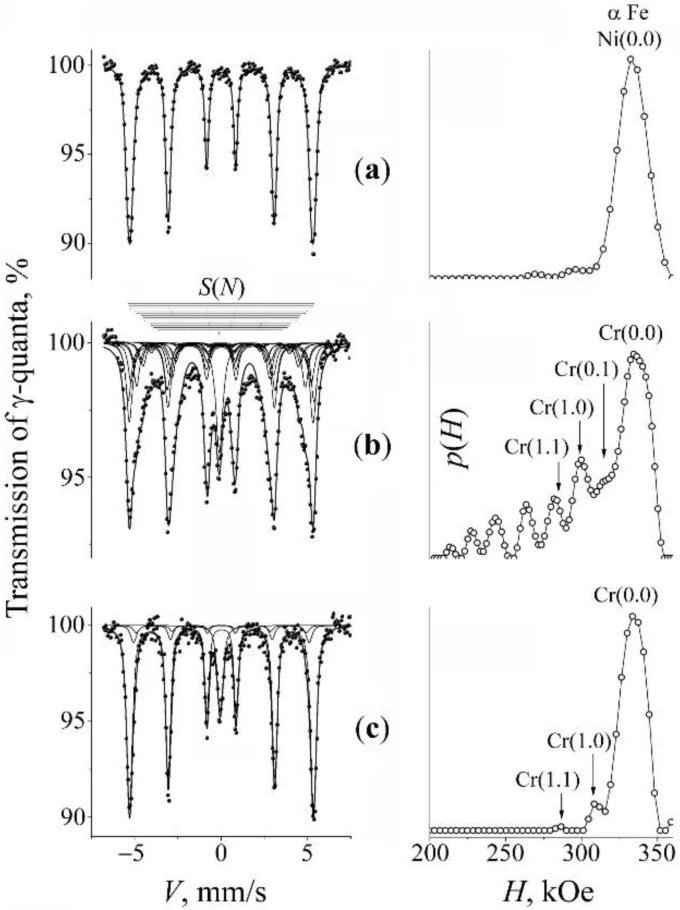
Mössbauer spectra of the mixture “C” (Fe–6Ni + CrN) and the distributions *p*(*H*) for the α-phase. Treatment: (**a**)—initial mixture; (**b**)—PBM, 10 h; (**c**)—PBM, 10 h + anneal 650 °C, 1 h. The arrows at the distribution *p*(*H*) mark the peaks corresponding to occupations by the impurity atoms Cr in the first two CSs of iron.

**Figure 4:**
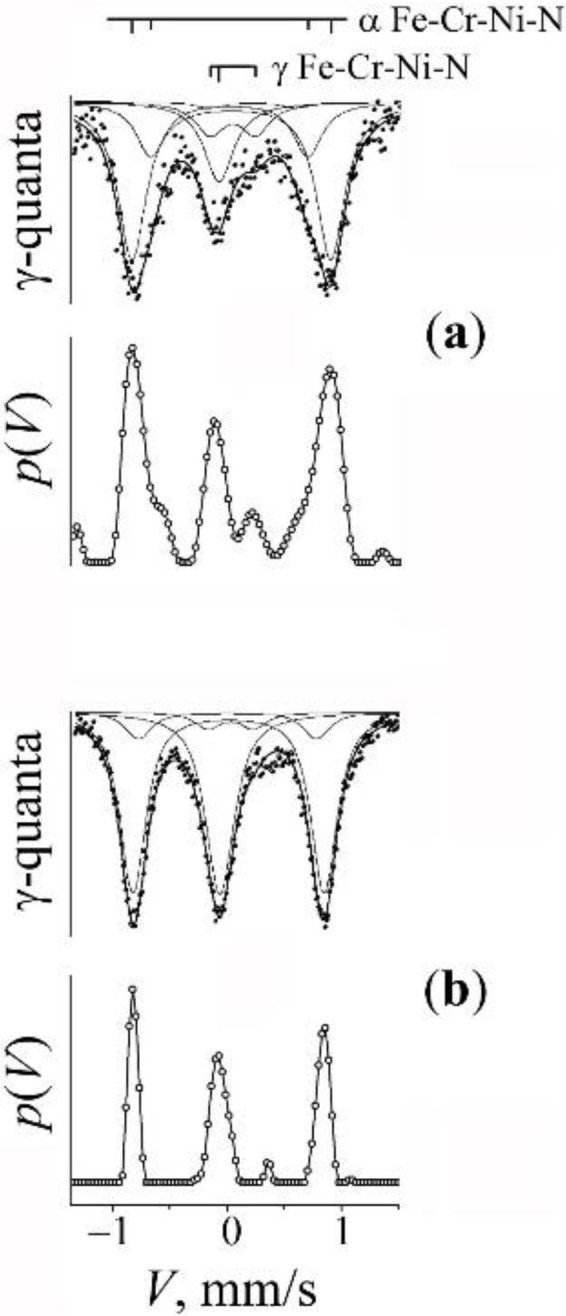
The central part of spectra for the mixture “C” (Fe–6Ni + CrN) and the distributions *p*(*V*). Treatment: (**a**)—PBM, 10 h; (**b**)—PBM, 10 h + anneal 650 °C, 1 h.

**Figure 5:**
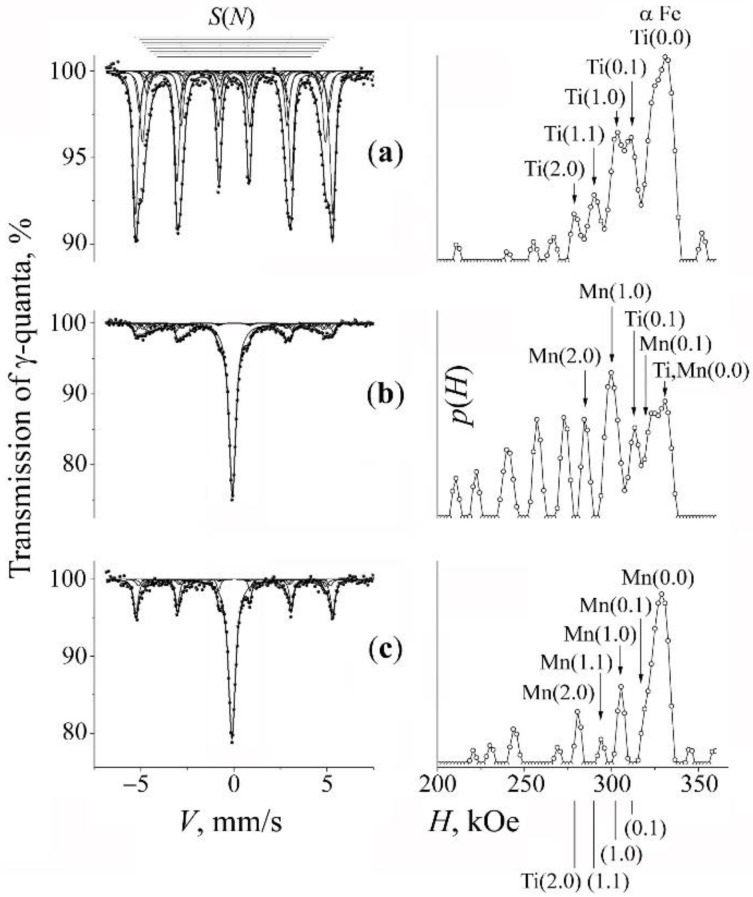
Mössbauer spectra of the mixture “D” (Fe–5Ti + MnN) and the distributions *p*(*H*) for the α-phase. Treatment: (**a**)—initial mixture; (**b**)—PBM, 10 h; (**c**)—PBM, 10 h + anneal 650 °C, 4 h. The arrows at the distribution *p*(*H*) mark the peaks corresponding to occupations by the impurity atoms Ti and Mn in the first two CSs of iron.

**Figure 6:**
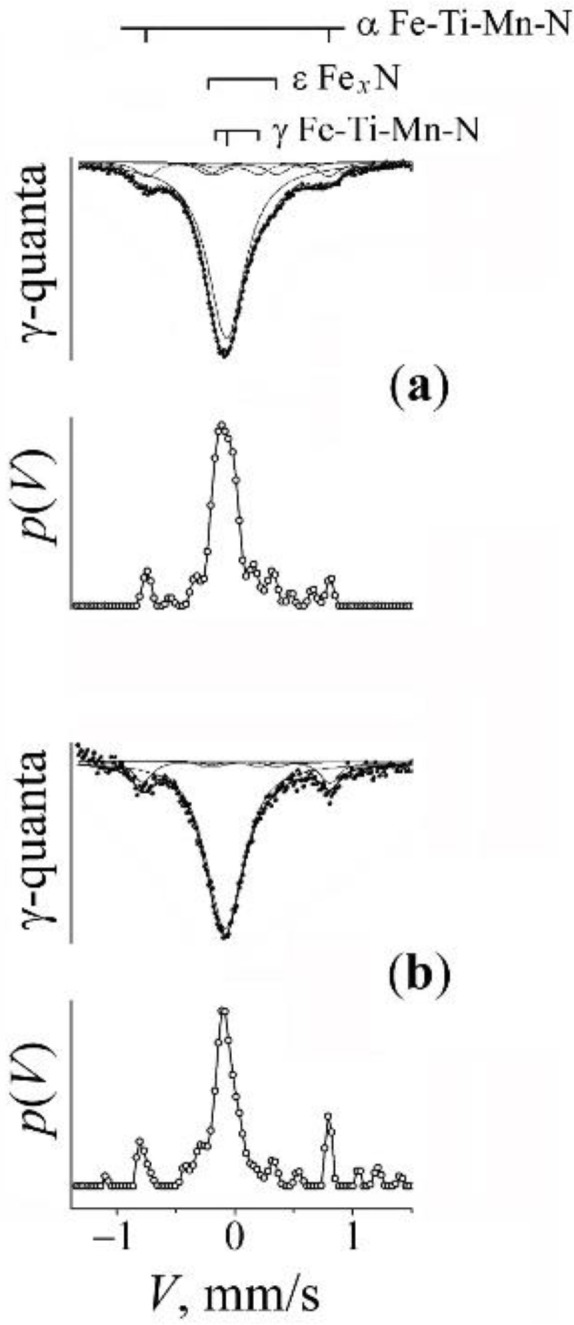
The central part of spectra for the mixture “D” (Fe–5Ti + MnN) and the distributions *p*(*V*). Treatment: (**a**)—PBM, 10 h; (**b**)—PBM, 10 h + anneal 650 °C, 4 h.

In Figure 1, Figure 2, Figure 3, Figure 4, Figure 5 and Figure 6, we present the experimental spectra and results of their calculation. The Mössbauer spectra of the MS samples are of multicomponent structure and include those typical of solid solutions in the α-ferrite and γ-austenite phases. Taking into account the complexity of the Mössbauer spectra, we used for their «calculation» the MS Tools application package [33]. In particular, the DISTRI program was employed, which is used in the case of locally inhomogeneous systems with a multicomponent structure and poor resolution of spectra. With its help, one can improve a resolution of spectra via the restoration of the distributions *p*(*V*) and *p*(*H*) probabilities of resonant absorption on the scale of the effective magnetic field *H* and Doppler velocities *V*, correspondingly. Further, based on the type of shape of the distributions *p*(*V*) and *p*(*H*) and on the results of analysis of the a priori data, we performed the choosing of the models of Mössbauer spectra. The parameters of the hyperfine structure and partial contributions of the components of the spectra were calculated using a standard procedure for approximating integral spectra by a superposition of components with Lorentz lines in the SPECTR program [33].

Spectra taken from the powders of the initial mixtures present by themselves sextets of the α-phase in the ferromagnetic alloys Fe–*x*Ni and Fe–*y*Ti, which has a BCC crystal lattice (see Figure 1a, Figure 3a and Figure 5a). The spectra of the α-phase of the MS samples have the form of sextets with broadened lines, which are a superposition of the *S*(*m*,*n*) subspectra (sextets) corresponding to the non-equivalent surroundings of resonant iron atoms formed by an admixture of chromium, nickel, titanium, and manganese chemical elements [34,35]. As a result of the mechanical synthesis and due to the formation of solid solutions, along with the appearance of the sextet from the ferromagnetic α-phase, in the center of the spectra one can observe the appearance of a broadened asymmetrical singlet of the paramagnetic γ-phase—austenite (see Figure 1b, Figure 3b, Figure 4a, Figure 5b and Figure 6a).

In the first approximation (without taking into account the difference in the Debye–Waller factors of phase components), the ratio of the areas under the integral sextet and a central singlet corresponds to the quantitative ratio (in volume percentages) between the α- and γ-phases.

The spectra from the ferritic α matrix in MS alloys and, in particular, the content of substitution impurities in it were calculated under the assumption of the additive contribution from the transition chemical elements Ni, Mn, Cr, and Ti to the change in the isomer shift of *I*_S_ and the effective field *H* on iron atoms in dilute solid solutions [34,35,36]. In accordance with this assumption, the change in the values of hyperfine parameters (*X*), namely, the isomer shift *I*_S_ and effective magnetic field (*H*), at the nucleus of Fe from the impurity of transition metals in the limits of the nearest coordination shells (CS) is described by the equation:

(1) $$X(m,n)=X(0,0)+m\cdot \Delta X_{1}+n\cdot \Delta X_{2},$$

where *X*(*m*,*n*) is the value of hyperfine parameter realized at the number of m atoms in the first CS and at n atoms of impurities in the second CS. The possibility of analyzing the mechanical alloying by the chromium, manganese and titanium substitution impurities of the metal matrix α-Fe solid solution is due to the difference in their effect on the hyperfine parameters of the nearest CSs of the resonant atom of iron [34] (see Table 2). The content of the substitution impurity was estimated by isolating the partial contributions (PCs) to the spectra from the atomic surroundings of resonant iron with an impurity, namely, the PCs of sextets *S*(*m*,*n*), where *m* and *n* are the numbers of impurity atoms in the first and second CSs of iron, respectively. The effective concentration of chemical elements of substitution was calculated according to the equation [36]:

(2) $$c_{1,2}=\frac{z_{1}\cdot c_{1}+z_{2}\cdot c_{2}}{z_{1}+z_{2}}$$

where $c_{1}=\frac{<m>}{z_{1}}$ and $c_{2}=\frac{<n>}{z_{2}}$ are the effective concentrations of the impurity in the first and second CSs of iron, respectively; <m>=∑m⋅W(m,n) and <n>=∑n⋅W(m,n) are the average number of the impurity atoms in the first and second CSs, respectively; and *z*_1_ and *z*_2_ are the coordination numbers for the first and second CSs of iron, respectively. The probabilities *W*(*m*,*n*) of finding the impurity are proportional to the integral intensities *S*(*m*,*n*) that were isolated from the experimental spectrum.

The occurrence of atoms of nitrogen in interstitial positions of a ferromagnetic matrix of the alloy can be represented in the form of additional subspectra from α-Fe–N, namely, those denoted as α-Fe, *A*, *A*′, and *B*, which are similar to the case of the spectrum from nitrided martensite [37].

The analysis and modelling of the spectrum from nitrided austenite is based at the a priori information on the spectrum from the stainless steel [38,39] and nitrided austenite [40,41]. In approximating the distribution *p*(*V*) via the employment of Gaussian forms, the model of spectrum can be represented in the form of the superposition of components *D*(0) + *D*(1) + *D*(2) (see Figure 4 and Figure 6 as well as Table 3). The expanded singlet *D*(0) corresponds to the atoms of the resonant iron without an impurity of nitrogen in the octahedral interstices of the first coordination shell (CS the singlet *D*(0) can be «restored» via the values of hyperfine parameters typical of stainless steel) [38,39]. The doublets *D*(1) and *D*(2) have hyperfine parameters (i.e., the isomer shift *I*_S_ and the quadrupole shift *Q*_S_), close in value to those of the parameters of the doublets from embedding one or two (dumbbell-like configurations of) atoms of nitrogen in the octahedral interstices of FCC iron [40,41].

The assessment of the nitrogen content (*c*_N_) in austenite of the MS samples was carried out under the assumption of repulsive distribution (mutual repulsion) of nitrogen atoms in a solid solution by the evaluation of the contribution of the configuration *D*(1) from iron atoms with one nitrogen atom in the nearest octahedral interstices, i.e., by the relative integral intensity *S_D_*_(1)_, similarly to that as in [15,19,40], in accordance with the equation [40]:

(3) $$S_{D(1)}=6\cdot p(1-p),$$

where $p=\frac{c_{N}}{1-c_{N}}$ is the fraction of the octahedral interstices in austenite «filled» by nitrogen. Equation (3) does not take into account the contribution from Mn and Cr atoms replacing the resonant ^57^Fe in austenite as a result of mechanical synthesis. However, it does not exceed 10 at.%, which can lead to a systematic error in estimating the nitrogen content by no more than 0.5%.

### 3.2. Mechanical Synthesis to Produce Fe–Cr–Ti–N Ferrite-Martensitic Steel

For the manufacturing of Fe–Cr–Ti–N ferrite-martensitic steel, we used the mixture «A» labeling the composition Fe–2Ti + 20% CrN. Figure 1 shows the spectra of the initial mixture (Figure 1a), the results of the PBM milling for 10 h (Figure 1b). and subsequent annealing at 500 °C, 4 h (Figure 1c). The Mössbauer spectrum of the initial mixture is a sextet characteristic of the α-Fe–2Ti solid solution. This is evidenced by peaks on the distribution *p*(*H*) and the subspectra *S*(*m*,*n*) corresponding to the positions of titanium in the next two CS relative to resonant iron (see [34] and Table 2). The calculation of the effective concentration using the partial contribution of *S*(*m*,*n*) indicates that an average concentration *c*_Ti_ of titanium in a solid solution was ~2.3 at.%.

The result of milling the mixture “A” for 5 and 10 h is the broadening of the sextet lines from the ferromagnetic α matrix and the appearance of additional peaks on the distribution *p*(*H*) in the value intervals of fields smaller than the α-Fe-related field (of 330 kOe) due to the ingress of chromium into the α solid solution (see Figure 1b and Table 2). The confirmation of the appearance of chromium in the position of substitution in the α solid solution is also the growth of the effective field of the impurity-free configurations *H*(0,0) from 331 to 335 kOe. The average value of the effective field <*H*> associated with that from the α-phase sextet decreases from 324 to 308 kOe. The estimation of the total content of the substitution impurity (Ti + Cr) after PBM processing, assessment based on the partial contribution from ***S***(0,0), and the assumption on the random distribution of Ti and Cr [21,36], amounts to 6.2 at.%. Apart from the contribution from the titanium and chromium impurities to the *p*(*H*) distribution, we have in *p*(*H*) the *A*′ component (with a characteristic field of ~345 kOe) that corresponds to the atomic configurations of the α interstitial solid solution of nitrogen atoms that occupy the octahedral interstices of the second coordination shell of resonant iron [37] (see Figure 1b).

The spectra and the distribution *p*(*H*) characteristic of the MS mixture “A” after its thermal annealing at 500 °C, for 4 h are shown in Figure 1c. According to the calculation data, it can be seen that the result of annealing is the preservation of chromium in the formed BCC solid solutions with a slight decrease in the total content of chromium impurity *c*_Cr_ to ~5%. At the same time, in the distribution *p*(*H*) of the annealed sample, there are no peak intensities from the titanium impurity in positions (1,0) and (0,1), which indicates the absence or significant decrease in the amount of titanium in the MS solid solution (see Figure 1c and Table 1). Thus, the result of the milling and subsequent annealing of the mixture “A” is the exit of titanium from the position of substitution of α solid solution and its replacement with chromium. There is also a decrease in the fraction of the component *A*′ corresponding to nitrogen in the interstitial position of a solid solution. The absence of visible peaks on the *p*(*H*) distribution characteristic of the α-Fe-Ti solution in the spectrum after the annealing of the MS alloy may indicate the release of titanium from the α solid solution and its ingress into secondary TiN nitrides.

In order to verify the effect of titanium impurity on the processes of mechanical alloying of the α matrix by chromium, experiments were performed on the mechanical alloying of mixtures not containing titanium: “B” (Fe + 20% CrN) and “C” (α-Fe–6Ni + 20% CrN). The spectra and *p*(*H*) of the initial mixtures and the results of the PBM processing, and subsequent annealing are presented in Figure 2 and Figure 3. It is obvious that the milling of the mixtures “B” and “C” leads to changes that are similar to those after the MS synthesis with the mixture “A”, which testifies to the appearance of chromium in the substitution positions of the α solid solutions, but in a lower amount (~4–5 at.%) then in the characteristic case of the composition Fe–2Ti + 20% CrN. In addition, a broadened asymmetric singlet corresponding to the paramagnetic γ-phase (7–10 vol.%) was formed in the center of the spectrum. The shape of the form of the singlet and its modelling in accordance with the model of repulsive distribution of nitrogen permits to estimate the amount of nitrogen atoms in the interstitial positions of austenite after milling as *c*_N_ ~8–9 at.% (see Figure 4a). Nitrogen doping of the α-phase manifests itself in the appearance of a peak *A*′ with a field of 348 kOe on the distribution *p*(*H*), corresponding to the position of nitrogen «incorporation» in the α solid solution of iron [37].

A subsequent annealing of the mixtures “B” and “C” leads predominantly to a return of the spectra corresponding to the α-phase right to the initial form typical of a moderate (up to 1%) content of chromium in the α solid solutions. In addition, in the mixture “B”, the singlet completely disappears, and in the mixture “C”, its intensity and the contribution of the “nitrogen” doublet *D*(1) in the austenite spectrum decreases (see Figure 4b and Table 1). Thus, in contrast to the case of the mixture with titanium, there is an almost complete release of chromium and nitrogen from the metal matrix of synthesized alloys.

### 3.3. Mechanical Synthesis of Fe–Mn–Ti–N Austenitic Steel

The spectrum characteristic of the initial mixture “D” having the composition Fe–5Ti + 20% Mn_2_N is described by the spectrum corresponding to the α-Fe–5Ti solid solution, which is similar to the spectrum from the initial mixture “A” (Figure 5a). The titanium content in the initial metal matrix was 5.2 at.%. The result of milling for 5 and 10 h is the appearance (in the integrated spectrum) of a broadened central singlet from austenite with the intensity of 70 vol.% and greater (see Figure 5b and Figure 6a). The central singlet has the typical form of nitrided austenite; of the latter, typical is the superposition of the components *D*(0), *D*(1), and *D*(2) (see Figure 6a and Table 3). The content of nitrogen in the γ solid solution amounts to ~1.3 at.%. In addition, there are lines in the central singlet, presumably of the ε-Fe*_x_*N nitride [42,43,44]. The confirmation of the “nitride” nature of the additional lines of the central doublet is the preservation of their intensity after annealing at 500 °C (see Figure 6b). In the spectrum from the α-phase, after milling, *S*(*m*,*n*) sextets appear typical of manganese in the nearest CS of iron, i.e., an α solid solution of Fe–Mn is formed (see Figure 5b). The effective concentration of the titanium and manganese in the α-phase of the MS alloy amounts to 1.5 and 10.3, respectively. The effective concentration of the impurities of substitution *c*_Ti + Mn_ amounts to ~12 at.%.

The subsequent anneals at 500 °C and 800 °C change little the ratio of the intensities of the α- and γ-phases (Figure 5c). In the spectrum of singlet from the γ-phase, a decrease in the intensity of the “nitrogen” doublet *D*(1) is observed (see Figure 6b and Table 3). The result of annealing is a narrowing of the α-phase sextet lines. On the distribution *p*(*H*), only the density peaks from manganese atoms in the nearest CSs of iron are preserved (see Figure 5). The manganese content in the α solid solution after annealing is 2.5 at.%.

**Figure 7:**
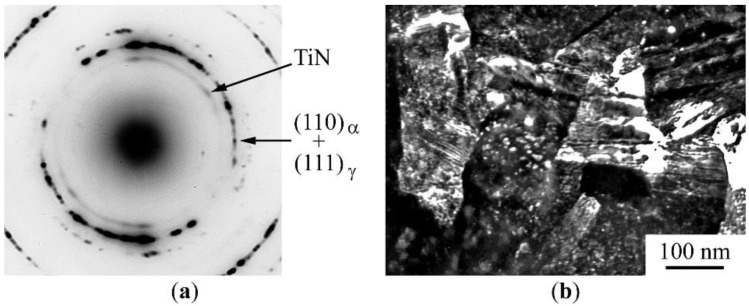
TEM data for the mixture “D” (Fe–5Ti + MnN) after PBM for 10 h and annealing at 650 °C, 4 h. (**a**)—AED pattern; (**b**)—dark-field image taken in the combined reflection (111)_TiN_ + (110)_α_ + (111)_γ_.

According to the transmission electron microscopy (TEM) data, a two-phase (α + γ) submicrocrystalline structure with a grain size of 120–250 nm was formed in an alloy of the composition “D” ((Fe–5Ti) + Mn_2_N) after the MS and annealing at 650 °C for 4 h. The corresponding micro-diffraction pattern contains ring reflections from strongly misoriented grains of martensite and austenite (Figure 7a). As a result of the MS and subsequent annealing, the particles of low-stability manganese nitride dissolved and secondary nanoparticles of more stable titanium nitride formed. The size of these particles amounted to ~2 nm. On the microdiffraction in front of matrix rings ((110)_α_ + (111)_γ_), there is a ring whose interplanar distance corresponds to the interplanar distance for planes of (111)_TiN_ type. On a dark-field image taken in a complex reflection ((111)_TiN_ + (110)_α_ + (111)_γ_)), both matrix elements and secondary nanoparticles of TiN nitrides are shined, which are evenly distributed over the grain body (Figure 7b).

## 4. Discussion

A feature of the proposed approach to obtain nitrogenous composites is the implementation of a scheme for the mechanically activated dissolution of low-strength nitrides in a metal matrix, followed by the precipitation of secondary hardening nitrides, in this case, heat-resistant titanium nitrides. The appearance of secondary nanocrystalline chromium nitrides during the mechanical activation of Fe-Ni(Mn, Cr) alloys with chromium and manganese nitrides that are poorly resistant to deformation was shown earlier in [17,19,21].

In Section 3.2 and Section 3.3, it was shown that the use of titanium as an alloying additive of iron makes it possible to accelerate the dissolution of primary CrN (Cr_2_N) and Mn_2_N nitrides and to preserve the elements Cr and Mn originated from nitrides in the positions of substitution of the metal matrix of MS alloys after thermal annealing. In the model mixtures “B” and “C”, such milling is accompanied by the less active dissolution of primary nitrides, and subsequent annealing leads to an almost complete release of the doping elements Cr and N from the α and γ solid solutions and to the destabilization of austenite.

The TEM data on the results of the MS mixture “D” confirm the results of the Mössbauer measurements on the formation of a two-phase (α + γ) structure with secondary stable disperse titanium nitrides. The preservation of the submicrocrystalline structure in the MS alloy during high-temperature annealing can be partly explained by the decomposition of solid solutions accompanying the phase α → γ transition with the formation of secondary TiN nitrides at the grain boundaries, which inhibit the process of the recrystallization growth of grains.

The data of the Mössbauer spectroscopy and TEM microscopy allow us to conclude that the direction and kinetics of the observed mechano-chemical reactions and structural-phase transitions depend on the physico-chemical properties of the alloying elements Ni, Fe, Mn, Cr, and Ti, which are part of the metal matrices. The elements Cr, Mn, Fe, and Ni belong to the VI–VIII groups of transition metals of the periodic table and have a reduced heat of nitride formation relative to titanium (group IV) at room temperature. The heat of formation of transition metal nitrides, as well as the melting point and the heat of sublimation of atoms, characterizes the stability of the position of atoms in the crystal lattice [4], and the heat of formation of nitrides decreases sequentially from titanium to chromium, then to iron and nickel, which apparently determines the binding energy of the corresponding chemical elements in nitrides. Thus, the parameters of the heat of nitride formation play an important role both in the conditions of the deformation-induced dissolution of primary nitrides (in this case, Mn_2_N and CrN) and the subsequent formation of secondary nitrides under milling and thermal-annealing conditions. The influence of the physico-chemical properties of the elements of mechanical mixtures on the direction and kinetics of mechanical alloying was shown earlier by the example of iron doping with nitride-forming vanadium and aluminum. In the MS experiment of Fe–V(Al) alloys with CrN nitrides, the substitution of V and Al for less stable Cr in nitrides with the formation of VN and AlN nitrides was established [15]. Similar experiments on deformation-induced red-ox reactions were performed during the dissolution of low-stability iron and copper oxides with the formation of secondary hardening oxides [22,23,24,25].

The condition for the active diffusion of the substitution elements (Ti, Cr, Fe, and Ni) and interstitial element (N) in the metal matrix of iron, leading to accelerated dissolution of disperse primary nitrides and subsequent decomposition of the formed α and γ solid solutions, is the saturation of the structure with linear and point defects [45]. In [20], this effect of the accelerated non-equilibrium dissolution of disperse nitrides and dynamic aging was shown on high-nitrogen chromium manganese steel Fe–22Mn–18Cr–0.8N under conditions of warm (100–300 °C) shear deformation under pressure in Bridgman anvils. At PBM processing, the process of dynamic aging with the release of titanium from the metal matrix manifests itself in a decrease in the effective concentration of titanium *c*_Ti_ from 2.5 at.% in the initial alloy down to 1.6 at.%, after the end of milling. At the same time, the total content of alloying elements, titanium and manganese, in the solid solution increases to 12 at.% (see Table 1). The acceleration of atomic mass transfer during the dissolution of primary dispersed nitrides and the formation of secondary nitrides is facilitated by the local temperature in the collision zone of the balls during milling processing [46].

The factors of the stabilization of the chemical and phase composition established during milling and subsequent annealing of nitrided α and γ solid solutions were described in [21]. It was shown that the main reason for the stabilization of austenite of the MS samples having the composition of Fe–6Cr + 20% Mn_2_N is the dissolution of Mn_2_N nitrides with the formation of inhomogeneous regions of the metal matrix enriched in austenite-forming manganese. In this article, the influence of the chemical activity of titanium on the acceleration of deformation-induced mechano-chemical reactions in a ball mill was established. These processes follow the path of dissolution of primary nitrites with the formation of Fe–Cr(Mn)–Ti–N solid solutions and the release of secondary extremely disperse titanium nitrides. It should be noted that the formation of a nitride-strengthened structure can be realized not only during milling in a ball mill, but also with other methods of severe plastic deformation, for example, high pressure torsion [20] or frictional action on the surface structure of alloys containing low-stability dispersed nitrides [17,47].

## 5. Conclusions

Structural-phase transformations during solid-phase mechanical synthesis (MS) in a ball mill of high-nitrogen ferrite-martensitic and austenitic steel composites were studied using Mössbauer spectroscopy and transmission electron microscopy. In mechanical synthesis, mixtures of iron alloys alloyed with transition metals and CrN (Cr_2_N) and Mn_2_N nitrides unstable under mechanical action were used. It was established that the use of titanium (which has the greatest heat of nitride formation) as an alloying additive of the Fe component of the mixture accelerates the processes of the dissolution of primary nitrides and provides the transferring of chromium and manganese to the position of substitution in the metallic solid solution. In addition, the titanium additive entails the formation of second-phase nitrides with stabilizing the nanostructure of MS samples. The condition of active diffusion of elements leading to mechanical alloying and synthesis is the saturation of the structure with linear and point defects. It is concluded that the binding (bond) forces between the atoms of transition metals and nitrogen affect the results on the phase and concentration composition in the observed mechano-chemical reactions. According to [4], the measure of bond forces in nitrides is the heat of the formation of nitrides involved in solid-phase mechanical synthesis.

Thus, the titanium alloying of iron in a mixture with CrN (Cr_2_N) nitrides makes it possible to obtain a high-nitrogen ferrite-martensitic composite, and in a mixture with Mn_2_N, a high-nitrogen austenitic composite stable during thermal annealing.

## Figures and Tables

**Table 2:** Magnitudes of magnetic hyperfine fields in alloys [34] in the initial mixture and the heat of the formation of nitrides with the chemical elements of alloying at room temperature [4].

Formula of Binary Alloys in Mixtures	Hyperfine Effective Fields *H*(*m*,*n*)			Formula of Nitride	Heat of Formation, kcal/g·atom
	*H*(0,0)	*H*(1,0)	*H*(0,1)		
Armco-Fe	330	–		Fe_4_N	–2.6
Fe–Ni	331	325	325	Ni_3_N	0.2
Fe–Mn	330	305	320	Mn_2_N	–48.2
Fe–Cr	335	304	311	CrN/Cr_2_N	–30/–31
Fe–Ti	330	306	314	TiN	−80.4

**Table 3:** Parameters of the hyperfine structure of the MS nitrided austenite obtained in the mixture “D”.

Treatment	Expanded Singlet *D*(0)			Doublet *D*(1)				Doublet *D*(2)		
	*I*_S_, mm/s	*G*, mm/s	*S*, %	*I*_S_, mm/s	*Q*_S_, mm/s	*G*, mm/s	*S*, %	*I*_S_, mm/s	*G*, mm/s	*S*, %
PBM, 10 h	–0.07	0.38	77.4	0.02	0.37	0.21	6.0	0.22	0.78	3.0
PBM, 10 h + anneal 500 °C	–0.08	0.38	82.6	0.02	0.36	0.20	2.5	0.20	0.76	1.1

## Data Availability

Not applicable.
